# Engineering of antimicrobial peptide Brevinin-1pl: arginine, lysine, and histidine substitutions enhance antimicrobial-anticancer efficacy with reduced cytotoxicity

**DOI:** 10.3389/fchem.2025.1579097

**Published:** 2025-05-19

**Authors:** Jingkai Wang, Fanli Zeng, Xiaoling Chen, Tao Wang, Lei Wang, Mei Zhou, Yangyang Jiang, Tianbao Chen, Yongfei Fang, Jinwei Zhang

**Affiliations:** ^1^ Department of Traditional Chinese Medicine, Chongqing General Hospital, Chongqing, China; ^2^ Natural Drug Discovery Group, School of Pharmacy, Queen’s University Belfast, Belfast, United Kingdom; ^3^ Diagnosis and Treatment Center of Integrated Chinese and Western Medicine, Chongqing General Hospital, Chongqing, China

**Keywords:** antimicrobial peptide, brevinin-1, drug-resistant, amino acid substitutions, anticancer activity, selectivity and cytotoxicity

## Abstract

**Introduction:**

Antimicrobial peptides (AMPs) are promising candidates for combating multidrug-resistant infections, but their clinical application is often limited by challenges such as poor selectivity and high cytotoxicity. This study aimed to optimize the therapeutic potential of brevinin-1pl, a frog-derived AMP with broad-spectrum antimicrobial and anticancer activities.

**Methods:**

Major experimental approaches encompassed antibacterial activity evaluation, hemolytic potential assessment, bactericidal rate determination via time-kill kinetics, SYTOX Green-based membrane integrity analysis, and MTT assays for anti-proliferative effects.

**Results:**

Substitutions with arginine (brevinin-1pl-2R and brevinin-1pl-5R) enhanced activity against Gram-positive bacteria but reduced efficacy against Gram-negative strains. Lysine substitution (brevinin-1pl-6K) decreased activity against Gram-positive bacteria due to reduced hydrophobicity. In contrast, histidine substitution (brevinin-1pl-3H) showed diminished activity against Gram-negative bacteria (e.g., MRSA MIC increased from 2 µM to 4 µM) but reduced hemolysis, indicating improved selectivity. Mechanistic studies using SYTOX green assays confirmed membrane disruption as a primary mode of action, while suggesting alternative mechanisms for Gram-positive *Enterococcus faecium* and Gram-negative *Escherichia coli*. The brevinin-1pl and its analogues demonstrated significant inhibitory efficacy against both MCF-7 breast cancer cells and H838 non-small cell lung cancer cells at a concentration of 10^–4^ M. Notably, brevinin-1pl-3H exhibited low cytotoxicity toward normal HaCaT cells despite its high hydrophobicity, suggesting potential for dermatological applications.

**Conclusion:**

These findings demonstrate that strategic amino acid substitutions can optimize the therapeutic potential of AMPs, offering a promising approach to develop peptides with enhanced efficacy and reduced clinical side effects.

## 1 Introduction

The emergence of drug-resistant bacteria has aroused people’s interest in antimicrobial peptides (AMPS) as a promising alternative to traditional antibiotics (Antimicrobial Resistance Collaborators, 2022; Prestinaci et al., 2015). Compared with traditional antibiotics, amp has several advantages, including lower possibility of drug resistance development and broader antibacterial activity (Lazzaro et al., 2020; Mhlongo et al., 2023; Shi et al., 2022). Among the antimicrobial peptides from frog secretions, the brevinin-1 family is a prominent example. Members of this family have demonstrated potent broad-spectrum antibacterial activity, and some showed significant cytotoxic effects on various human cancer cell lines, positioning them as potential candidates for novel therapeutic agents (Jiang et al., 2020; Richter et al., 2022; Xiong et al., 2021). However, natural amps often face limitations, such as hemolytic activity and cytotoxicity (Zou et al., 2023). Therefore, the strategy of reducing these side effects while maintaining their biological activity has become the main focus of peptide modification research. In this case, amino acid substitution is one of the most widely explored methods.

The distinction between Gram-positive and Gram-negative bacteria is prioritized in clinical microbiology and therapeutic research due to their divergent structural architectures, resistance mechanisms, and public health impacts (De Oliveira et al., 2020). Gram-positive pathogens, characterized by a thick peptidoglycan layer, are often targeted by antibiotics disrupting cell wall synthesis, whereas Gram-negative bacteria possess an impermeable outer membrane rich in lipopolysaccharides, conferring intrinsic resistance to many antimicrobials and triggering severe inflammatory responses (Ruhal and Kataria, 2021). Both groups encompass high-priority pathogens (e.g., *Staphylococcus aureus* and *Pseudomonas aeruginosa* within the ESKAPE consortium) that drive life-threatening infections and exhibit escalating multidrug resistance (Gardete and Tomasz, 2014; Pang et al., 2019). The broad-spectrum antibacterial activity of antimicrobial peptides provides a new direction for the study and treatment of drug-resistant bacteria.

Arginine and lysine, which carry a net positive charge at physiological pH, are essential components of AMPs, as they mediate electrostatic interactions with negatively charged microbial membranes, leading to the disruption of membrane integrity (Sani and Separovic, 2016). This disruption leads to membrane permeation ultimately microbial cell death. Furthermore, the intrinsic multifunctional attributes of arginine and lysine facilitate the design of innovative AMPs characterized by enhanced therapeutic efficacy and reduced propensity for the emergence of resistance (Agrillo et al., 2023). There are also alpha-helical structured peptides that achieve stronger antibacterial activity and better hemolytic activity by replacement of the arginine (Yang et al., 2018).

Besides arginine and lysine, histidine also contributes an electrical charge under specific conditions. Histidine exists in the amino-acid sequences of AMPs in numerous marine organisms (Xian et al., 2022). It enhances the antimicrobial and anticancer effectiveness of AMPs through multiple mechanisms. A key characteristic of histidine is its role as a pH sensor, attributed to its imidazole side chain, which becomes protonated and carries a charge under acidic conditions but remains neutral at higher pH levels. This pH-dependent behavior enables histidine to adapt to varying environmental conditions, a feature that is particularly advantageous in microbial environments and biological systems where pH fluctuations are common (Kacprzyk et al., 2007).

The role of charge in the activity of AMPs is a central focus in antimicrobial research. AMPs are widely recognized for their ability to target a broad spectrum of pathogens, a property largely driven by their charge density. The positive charge of AMPs is critical for their interaction with microbial membranes, as the electrostatic attraction between the cationic peptides and the anionic components of microbial membranes enables initial binding, either to specific recognition sites or through non-specific adhesion (Henderson et al., 2016; Mesa-Galloso et al., 2021; Wang et al., 2017). This interaction disrupts the membrane’s structural integrity, leading to permeabilization and ultimately inducing microbial cell death.

Despite their considerable therapeutic potential, the clinical application of AMPs remains significantly constrained by several persistent challenges. These include dose-limiting toxicity (particularly hemolytic activity against mammalian cells), susceptibility to proteolytic degradation by endogenous enzymes, poor structural stability under physiological conditions, unfavorable pharmacokinetic profiles, and high production costs (Zeng et al., 2021). Notably, LL-37, the sole human cathelicidin AMP, exhibits a paradoxical dual role in immune regulation. While it serves as a potent bactericidal agent through membrane disruption and immunomodulation, its pathological overexpression in various inflammatory disorders (e.g., psoriasis) has been mechanistically linked to exacerbated autoimmunity (Herster et al., 2020; Macleod et al., 2019).These multifaceted limitations continue to restrict their widespread clinical adoption and therapeutic utility. Among AMPs, brevinin-1pl is particularly promising due to its broad-spectrum resistance activity and rapid bactericidal ability.

The rationale for investigating AMPs in this study is anchored in their unique evolutionary advantages and unmet therapeutic potential amid the global antimicrobial resistance crisis. Unlike conventional antibiotics that target specific molecular pathways (e.g., β-lactams inhibiting cell wall synthesis), AMPs exert multimodal antimicrobial actions primarily through rapid membrane disruption and immunomodulation which impose high evolutionary barriers to bacterial resistance development (Kawai et al., 2023; Yang et al., 2024). Building on our previous discovery of brevinin-1pl, a novel brevinin-1 peptide isolated from *Rana pipiens* (Wang et al., 2024).This study systematically examines the functional consequences of substituting arginine, lysine, and histidine residues within its sequence. Based on structural predictions and empirical insights, four analogs were obtained through targeted substitutions of arginine, histidine, and lysine residues. The experimental evaluation revealed that arginine substitutions in brevinin-1pl-2R and brevinin-1pl-5R enhanced hemolytic activity and accelerated bactericidal kinetics, highlighting the dual functional role of arginine residues. In contrast, lysine substitution in brevinin-1pl-6K reduced hemolytic activity, suggesting its utility in decreasing hemolysis. Notably, histidine-substituted brevinin-1pl-3H demonstrated potent antifungal activity while concurrently lowering hemolysis, indicating its potential for selective antimicrobial applications. These findings underscore the critical influence of specific residue substitutions on balancing therapeutic efficacy and safety in peptide design. In summary, the modification work in this study provided valuable ideas for the study of brevinin-1 peptide.

## 2 Materials and methods

### 2.1 Bioinformatics analysis

The helical wheel diagrams of peptides were generated using the HeliQuest server (https://heliquest.ipmc.cnrs.fr/) (Gautier et al., 2008). Parameters used: Helix type: α; Window size: Full; One-letter code size proportional to amino acid volume: no; Rotation of the helix to align vertically the <µH> vector: no. The secondary structures of the peptides were predicted utilizing the online server PEPFOLD4. (https://bioserv.rpbs.univ-paris-diderot.fr/services/PEP-FOLD4/) (Rey et al., 2023). The prediction results were visualized using the PyMOL program. Parameters used: Generator: fbt; Number of models:100; Monte Carlo steps: 30000; Monte Carlo temperature: 370; Pseudo-random seed: 1.

### 2.2 Peptide synthesis and characterization

The peptides utilized in this study were synthesized through the solid-phase peptide synthesis (SPPS) method, employing a Tribute peptide synthesizer (Gyros Protein Technology, United States). Following synthesis, all peptides were purified using a reverse-phase high-performance liquid chromatography (RP-HPLC) system (Waters, United Kingdom) fitted with an Aeris Peptide XB-C18 HPLC column (250 × 21.2 mm, 5 µm, Phenomenex, United Kingdom), purity threshold: ≥95%. The purified peptides were subsequently characterized using matrix-assisted laser desorption/ionization time-of-flight (MALDI-TOF) mass spectrometry (4800 MALDI TOF/TOF, Applied Biosystems, USA). Observed mass within ±50 ppm of theoretical mass.

### 2.3 Secondary structure determinations and analysis

The secondary structures of the peptides were analyzed using circular dichroism (CD) spectrometry (J-815, Jasco, United Kingdom). For the analysis, the peptides were prepared at a final concentration of 50 µM in 10 mM NH_4_AC to simulate aqueous environments and 50% trifluoroethanol (TFE, v/v, in 10 mM NH_4_AC) to mimic membrane-like environments. The path length of the cuvette range was set from 190 nm to 260 nm, with a scanning speed of 100 nm/min. The bandwidth and data pitch were configured at 1 nm and 0.5 nm, respectively. The BeStSel (Beta Structure Selection) server was utilized to analyze the possible secondary structure contents of peptides in different environments (https://bestsel.elte.hu/index.php) (Micsonai et al., 2022).

### 2.4 Antimicrobial assays

The minimal inhibitory concentration (MIC) and minimal bactericidal concentration (MBC) values of the peptides were determined by established protocols to evaluate their antimicrobial activities (Lin et al., 2021). The MIC value was the lowest concentration of a peptide that inhibits visible bacterial growth after incubating 18 h (CLSI standard). Once the MIC values were determined, an aliquot from the bacterial culture exposed to the peptide at or above its MIC value was inoculated on a Mueller Hinton agar (MHA) and was incubated at 37 for 18 h (CLSI standard). The MBC value was the lowest concentration of a peptide that kills 99.9% of bacteria. Antimicrobial activities of synthetic peptides were assessed by determination of MIC and MBC values using eight kinds of bacteria and yeast: Gram-positive bacteria, *Staphylococcus aureus* ATCC 6538 (*S. aureus* 6538), Methicillin-resistant *Staphylococcus aureus* NCTC 12493 (MRSA 12493), and *Enterococcus faecalis* NCTC 12697 (*E. faecium* 12697); Gram-negative bacteria, *Escherichia coli* ATCC 8739 (*E. coli* 8739), *Pseudomonas aeruginosa* ATCC CRM 9027 (*P. aeruginosa* 9027), *Klebsiella pneumoniae* ATCC 43816 (*K. pneumoniae* 43816), and *Acinetobacter baumannii* BAA 747 (*A. baumannii* 747); and pathogenic yeast, *Candida albicans* ATCC 10231 (*C. albicans* 10231).

### 2.5 Hemolysis assays

The hemolytic activity of the peptides was assessed using horse erythrocytes. Horse erythrocytes were selected due to their similarity to human erythrocytes in membrane composition and widespread use in AMP studies for standardization. Following a previously established protocol (Gao et al., 2016). Peptide solutions with final concentrations ranging from 1 µM to 128 µM were incubated with 2% horse erythrocytes at 37°C for 2 h. Triton X-100 (1%) served as the positive control, while sterile PBS was used as the blank control. After that, the peptide-horse erythrocytes mixture was centrifuged at 930 *g* for 10 min, and the absorbance at 570 nm of the supernatant was measured. The hemolysis rate was calculated using the formal:

Percentage Hemolysis = (Absorbance of sample − Absorbance of blank)/(Absorbance of Positive- Absorbance of Blank) × 100%

### 2.6 Time-killing kinetic assays

Time-killing analysis monitors the effect of different concentrations of antimicrobials on bacterial growth stages over time. Bacteria that were applied to this experiment included Gram-positive bacteria, *S. aureus* 6538, MRSA 12493, and *E. faecium* 12697. The bacteria were subcultured for antimicrobial assay. MIC, two-fold MIC, and four-fold MIC of peptides were used and mixed with 5 × 10^5^ concentrations of bacteria. Samples of bacteria were removed at several time points (0 min, 5 min, 10 min, 20 min, 30 min, 60 min, 90 min, 120 min and 180 min). After the samples were incubated overnight, the bacterial concentrations at different time points were calculated by counting the number of colonies on the agar plates.

### 2.7 Sytox green permeability assays

Sytox green assays were used to evaluate the ability of cells to affect membrane permeability, Bacteria that were used in these experiments included Gram-positive bacteria, MRSA 12493, *E. faecium* 12697, and Gram-negative bacteria, *E. coli* 8739 and *K. pneumoniae* 43816, following a previously established protocol (Yin et al., 2024). Bacterial strains were grown to the logarithmic phase (Bacterial density: 1 × 10^6^ CFU/mL) and washed thrice with 30 mL of 5% TSB/0.85% NaCl via centrifugation at 1000 *g* for 10 min. Subsequently, 50 μL of bacterial suspension, 40 μL of peptide solution, and 10 μL of 1% SYTOX green-fluorescent nucleic acid stain (ThermoFisher, United States) were combined and incubated for 2 h at 37°C. In this assay, bacteria resuspended in 5% TSB/0.85% NaCl served as the growth control, while sterile PBS as the vehicle control. Fluorescence intensity was quantified using a Synergy HT microplate reader with excitation at 360 nm and emission detection at 460 nm.

### 2.8 MTT assays

Lung cancer cell line, H838 (ATCC, United States), breast cancer cell line, MCF7 (ATCC, United States), and human immortalized keratinocytes, HaCat (Caltag Medsystems, United Kingdom), were used in this experiment to test the anti-proliferation activity of peptides brevine-1pl and its four analogues. H838 cells were maintained in RPMI-1640 medium (Gibco, United Kingdom), whereas MCF-7 and HaCaT cells were cultured in DMEM medium (Gibco, United Kingdom), supplemented with 10% (v/v) fetal bovine serum (FBS, Gibco, United Kingdom) and 1% (v/v) penicillin-streptomycin (PS, Gibco, United Kingdom). Cells were seeded in 96-well plates at densities of 8,000 cells/well for H838 and MCF-7 and 10,000 cells/well for HaCaT, followed by overnight incubation at 37°C under 5% CO_2_ to allow adherence. Following attachment, cells were exposed to peptide solutions at final concentrations ranging from 10^–4^ M to 10^–9^ M. After 22 h of treatment, 0.5 mg/ml MTT solution was added to each well and incubated at 37°C for 4 h. The medium was then aspirated, and 100 μl of DMSO was added to solubilize formazan crystals. Plates were agitated for 15 min in a shaking incubator, and absorbance was measured at 570 nm using a Synergy HT microplate reader. Three biological replicates, each with three technical replicates (total n = 9), as stated in Results. Cell viability (%) was calculated using the formula: Viability (%) = (OD_treated_ − OD_blank_)/(OD_control_− OD_blank_) *100. OD_control_, OD_treated_ and OD_blank_ represent the optical density values of untreated controls, experimental samples, and blank wells, respectively. For IC50 determination, a linear dose-response relationship allows direct interpolation of the drug concentration corresponding to 50% inhibition. However, nonlinear dose-response curves require log-transformation of concentrations followed by nonlinear regression analysis using a model Y = Bottom + (Top - Bottom)/(1 + 10^((LogEC50-X) *HillSlope)^ in GraphPad Prism, which iteratively fits the data to derive IC50 values with 95% confidence intervals.

### 2.9 Statistical analysis

Experiment data in this work was analyzed using the GraphPad Prism 10.1.2 software (GraphPad Software Inc., San Diego, CA, United States), and the results are represented as standard error of the mean (S.E.M.). Significance levels were determined through one-way ANOVA tests, comparing the mean values of the designated data sets. Asterisks denote significant differences (*p < 0.05; **p < 0.01; ***p < 0.001; ****p < 0.0001). Tukey-Kramer Significant Difference (HSD) was used as *post hoc* test.

## 3 Results

### 3.1 Secondary structure prediction and modification of brevinin-1pl and analogues

**FIGURE 1:**
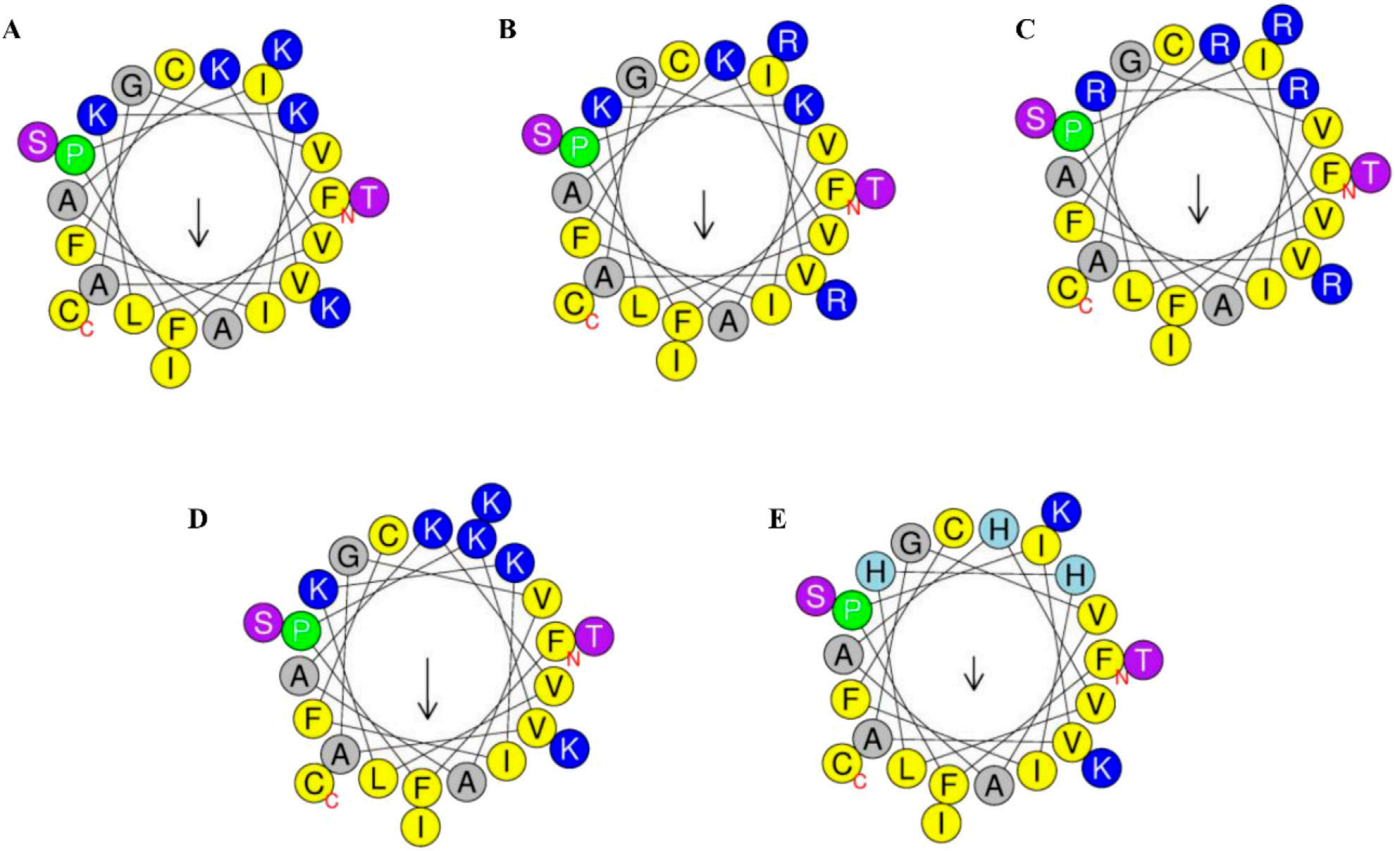
Helical wheel projection of the 24 amino acids of brevinin-1pl **(A)** /brevinin-1pl-2R **(B)**, brevinin-1pl-5R **(C)**, brevinin-1pl-6K **(D)** and brevinin-1pl-3H **(E)** predicted by HeliQuest. In the schematic representation, the color-coded amino acids are categorized based on their physicochemical properties: yellow residues denote non-polar amino acids, while grey residues indicate polar amino acids. Green residues specifically represent proline, which serves as the initiation point of the alpha-helical structure. Blue residues correspond to amino acids bearing an electrical charge. The arrow illustrates the orientation of the hydrophobic surface, with its directionality indicating the spatial orientation of hydrophobicity and its magnitude reflecting the relative extent of hydrophobic character.

Secondary structure analysis revealed that brevinin-1pl possesses a hydrophobic face and contains five lysine residues (as shown in Figure 1A). This study focused on amino acid substitution at the non-hydrophobic surface to investigate its impact on peptide properties. The theoretical molecular weight, net charge, hydrophobicity, and hydrophobic moment of the designed sequences are summarized in Table 1. To explore the effects of substitution, we first replaced the lysine residues at positions 22 and 23 with arginine to generate brevinin-1pl-2R (as shown in Figure 1B). Subsequently, all lysine residues (at positions 11, 14, 15, 22, and 23) were substituted with arginine to yield brevinin-1pl-5R (as shown in Figure 1C). Furthermore, brevinin-1pl-6K (as shown in Figure 1D) was created by replacing the isoleucine at position 4 with lysine. Finally, brevinin-1pl-3H (as shown in Figure 1E) was obtained by substituting lysine residues at positions 11, 14, and 15 with histidine, enabling an investigation into the structural and functional influence of histidine incorporation.

**TABLE 1:** Partial structural parameters of brevinin-1pl and its analogues.

Peptide name	Sequence	Theoretical molecular weight	Net charge	Hydrophobicity <H>	Hydrophobic moment <µH>
brevinin-1pl	FFPIVAGVAAKVLKKIFCTISKKC	2609	5	0.672	0.359
brevinin-1pl-2R	FFPIVAGVAAKVLKKIFCTISRRC	2666	5	0.670	0.359
brevinin-1pl-5R	FFPIVAGVAARVLRRIFCTISRRC	2750	5	0.668	0.361
brevinin-1pl-6K	FFPKVAGVAAKVLKKIFCTISKKC	2624	6	0.556	0.464
brevinin-1pl-3H	FFPIVAGVAAHVLHHIFCTISKKC	2637	2	0.812	0.253

NOTE: Red color letters represent the modified amino acids, while hydrophobicity generally represents the lipophilicity of AMPs, Hydrophobic moment generally represents the amphiphilicity of AMPs.

**FIGURE 2:**
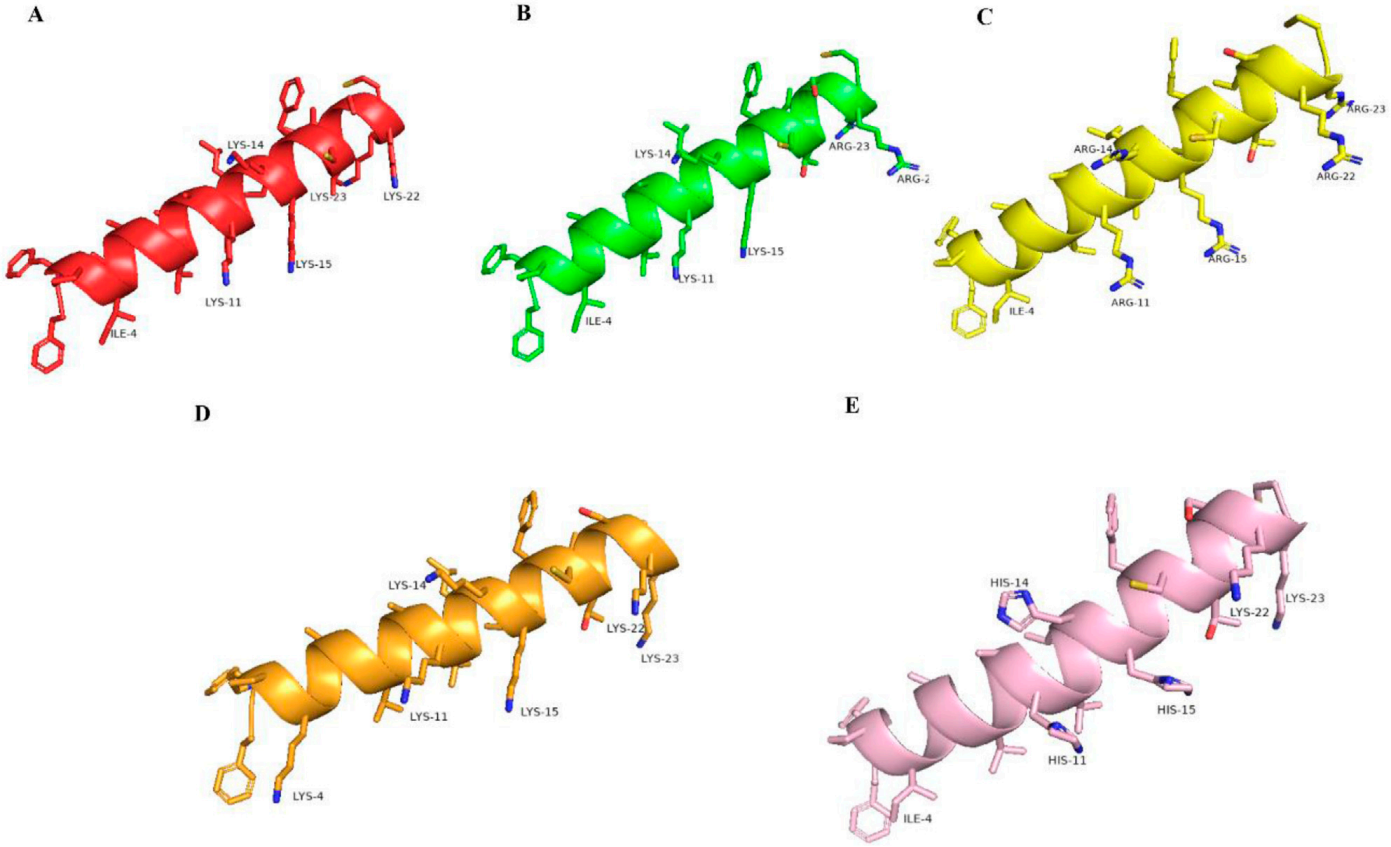
brevinin-1pl **(A)**, brevinin-1pl-2R **(B)**, brevinin-1pl-5R **(C)**, brevinin-1pl-6K **(D)** and brevinin-1pl-3H **(E)** predicted by Pepfold-4.

The structural prediction results of brevinin-1pl and its analogues obtained using the Pepfold-4 prediction software are shown in Figure 2. In the predicted results, the secondary structures of the modified peptides, including brevinin-1pl-2R (Figure 2B), brevinin-1pl-5R (Figure 2C), brevinin-1pl-6K (Figure 2D), and brevinin-1pl-3H (Figure 2E), are largely consistent with that of brevinin-1pl (Figure 2A), maintaining an alpha-helix conformation. Specifically, for brevinin-1pl-2R and brevinin-1pl-5R, the charge positions of their side chains remain unchanged. For brevinin-1pl-6K, the addition of lysine at position 4 results in a side chain orientation similar to that of other lysine residues in the sequence. Notably, brevinin-1pl-3H exhibits three distinct imidazole rings, with charges in a neutral environment originating exclusively from positions 23 and 24.

### 3.2 Secondary structure analysis of brevinin-1pl and its analogues

**FIGURE 3:**
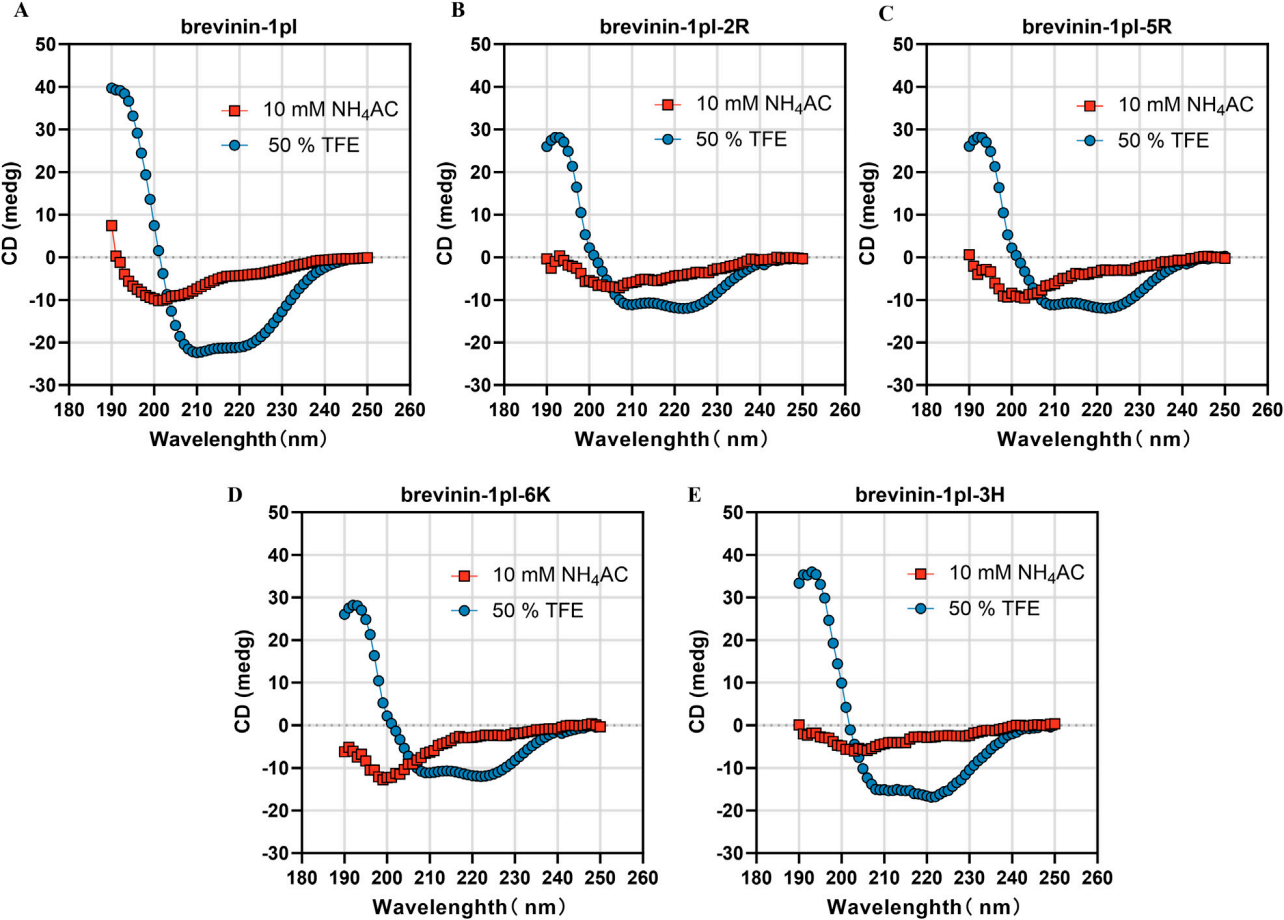
CD spectrum of brevilin-1pl **(A)**, brevilin-1pl-2R **(B)**, brevilin-1pl-5R **(C)**, brevilin-1pl-6K **(D)**, and brevilin-1pl-3H **(E)**. The red line represents the results of peptides in 10 mM NH4AC solution. The blue line represents the results of peptides in 50% TFE buffer.

The secondary structure of brevinin-1pl and its analogues was analyzed by using CD spectroscopy (Figure 3). In the 10 mM NH_4_AC solution, all peptides primarily adopt a random coil structure, while they mainly adopt an alpha-helix structure in the 50% TFE solution (Table 2). The replacement of Arg and His and the introduction of Lys had negligible effects on the helix content of brevinin-1pl (Figure 3A), brevinin-1pl-2R (Figure 3B), brevinin-1pl-5R (Figure 3C), brevinin-1pl-6K (Figure 3D) and brevinin-1pl-3H (Figure 3E). The α helix structure content of brevinin-1pl and its analogues is around 95%.

**TABLE 2:** Estimated α helix structure content (%) of brevinin-1pl and its analogues.

α helix (%)		
Peptide	NH4AC	TFE
brevinin-1pl	17.8	95.2
brevinin-1pl-2R	17.4	95.3
brevinin-1pl-5R	12.18	95.3
brevinin-1pl-6K	5.68	95.2
brevinin-1pl-3H	8.59	95.4

### 3.3 Antimicrobial activities of brevinin-1pl and its analogues

The antimicrobial activities of brevinin-1pl and its analogues, were screened by using eight types of microorganisms. The results of antimicrobial assays are shown in Table 3. Brevinin-1pl-2R exhibited antimicrobial activity comparable to that of the parent peptide, but its MBC against *P. aeruginosa* decreased from 16 to 8 μM. Brevinin-1pl-5R demonstrated reduced antimicrobial activity against Gram-negative bacteria, although its MIC against the Gram-positive bacterium *E. faecium* decreased from 4 to 2 μM. *Brevinin-1pl-6K* exhibited reduced antimicrobial activity against Gram-positive bacteria, but its activity against Gram-negative bacteria such as *E. coli* and *K. pneumoniae* remained consistent with that of the parent peptide. Finally, brevinin-1pl-3H, with the introduction of three histidine, exhibited antifungal activity at a concentration of 64 μM. While its bactericidal activity against Gram-negative bacteria was significantly reduced, it retained antimicrobial activity against Gram-positive bacteria, such as MIC of 4 μM against MRSA.

**TABLE 3:** Antimicrobial screening results of brevinin-1pl and its analogues. MICs/MBCs (µM) values are listed.

Strains	*S. aureus* 6538	MRSA 12493	*E. faecium* 12697	*E. coli* 8739	*K. pneumoniae* 43816	*P. aeruginosa* 9027	*A. baumannii* 747	*C. albicans* 10231
brevinin-1pl	2/2	2/2	4/4	4/4	8/8	8/16	8/16	>128/>128
brevinin-1pl-2R	2/2	2/2	4/4	4/4	8/8	8/8	8/16	>128/>128
brevinin-1pl-5R	2/2	2/2	2/2	8/8	16/16	16/16	16/32	>128/>128
brevinin-1pl-6K	4/4	4/4	16/16	4/4	8/8	8/8	16/16	>128/>128
brevinin-1pl-3H	8/8	4/4	16/32	32/32	32/64	64/64	>128/>128	64/>128

### 3.4 Hemolysis activities

**FIGURE 4:**
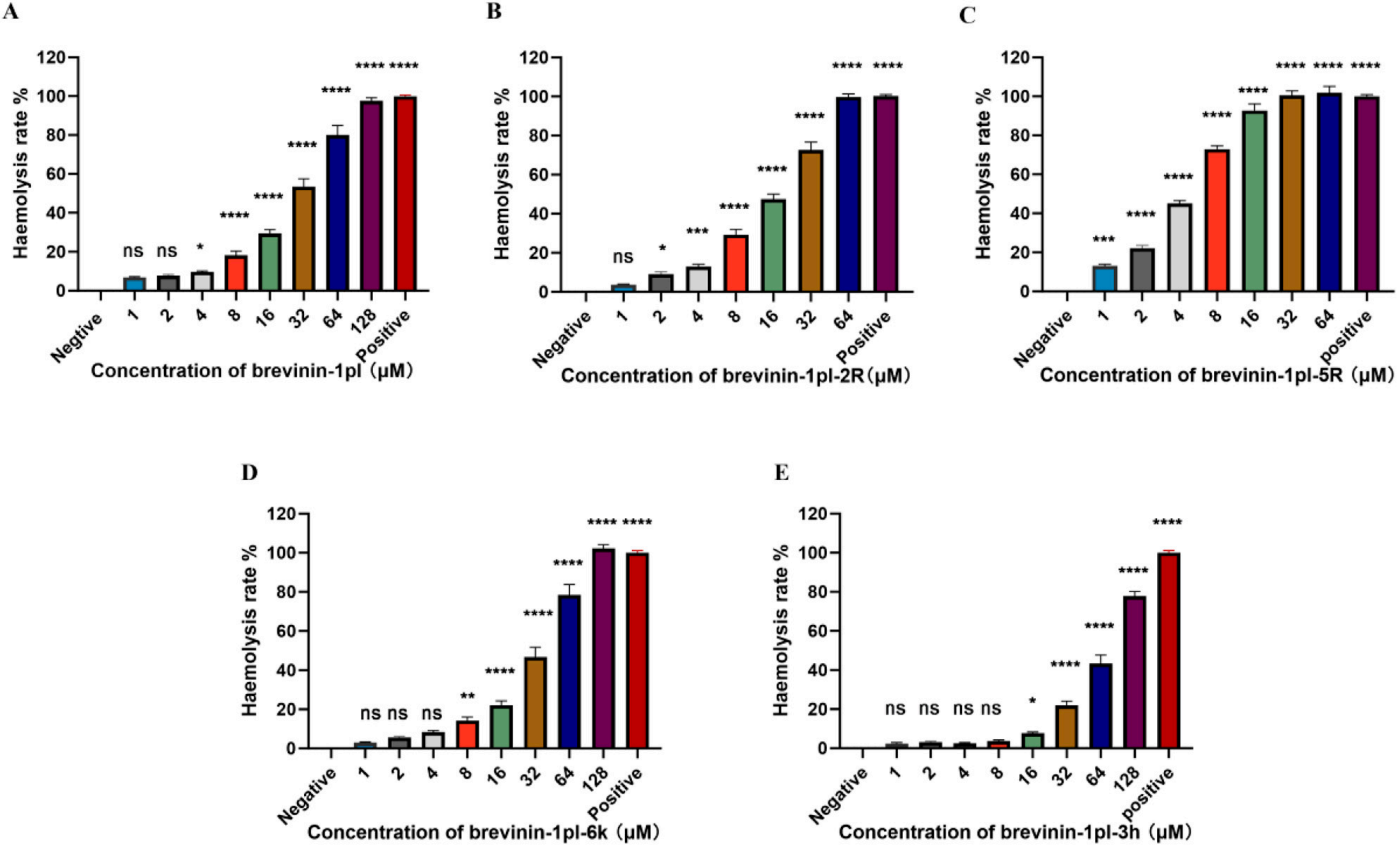
The hemolytic activities of peptide brevinin-1pl **(A)**, brevinin-1pl-2R **(B)**, brevinin-1pl-5R **(C)**, brevinin-1pl-6K **(D)**, and brevinin-1pl-3H **(E)** brevinin-1pl were evaluated using horse erythrocytes at peptide concentrations from 1 μM to 128 μM. The error bars represent the means ± SEMs for each independent experiment. Asterisks denote statistical significance compared to the 0.5% DMSO control group: *P < 0.05, **P < 0.01, ***P < 0.001, ****P < 0.0001. The P-value represents the statistical significance of the observed differences.

Hemolytic activity analysis revealed that compared with brevinin-1pl (Figure 4A) brevinin-1pl-2R (Figure 4B) and brevinin-1pl-5R (Figure 4C) exhibited increased hemolytic activity with the augmentation of arginine substitution, and hemolytic activity was already observed at their antimicrobial concentrations. Although brevinin-1pl-6K (Figure 4D) showed reduced antibacterial activity against Gram-positive bacteria, its MIC value against Gram-negative bacteria *E. coli* remained unchanged at 4 μM, and its hemolytic activity was also reduced. Brevinin-1pl-3H (Figure 4E) demonstrated inhibitory effects on three Gram-positive bacteria at 16 μM, while showed low hemolytic activity at this concentration.

### 3.5 Time-killing kinetics study of brevinin-1pl, brevinin-1pl-2R and brevinin-1pl-5R

**FIGURE 5:**
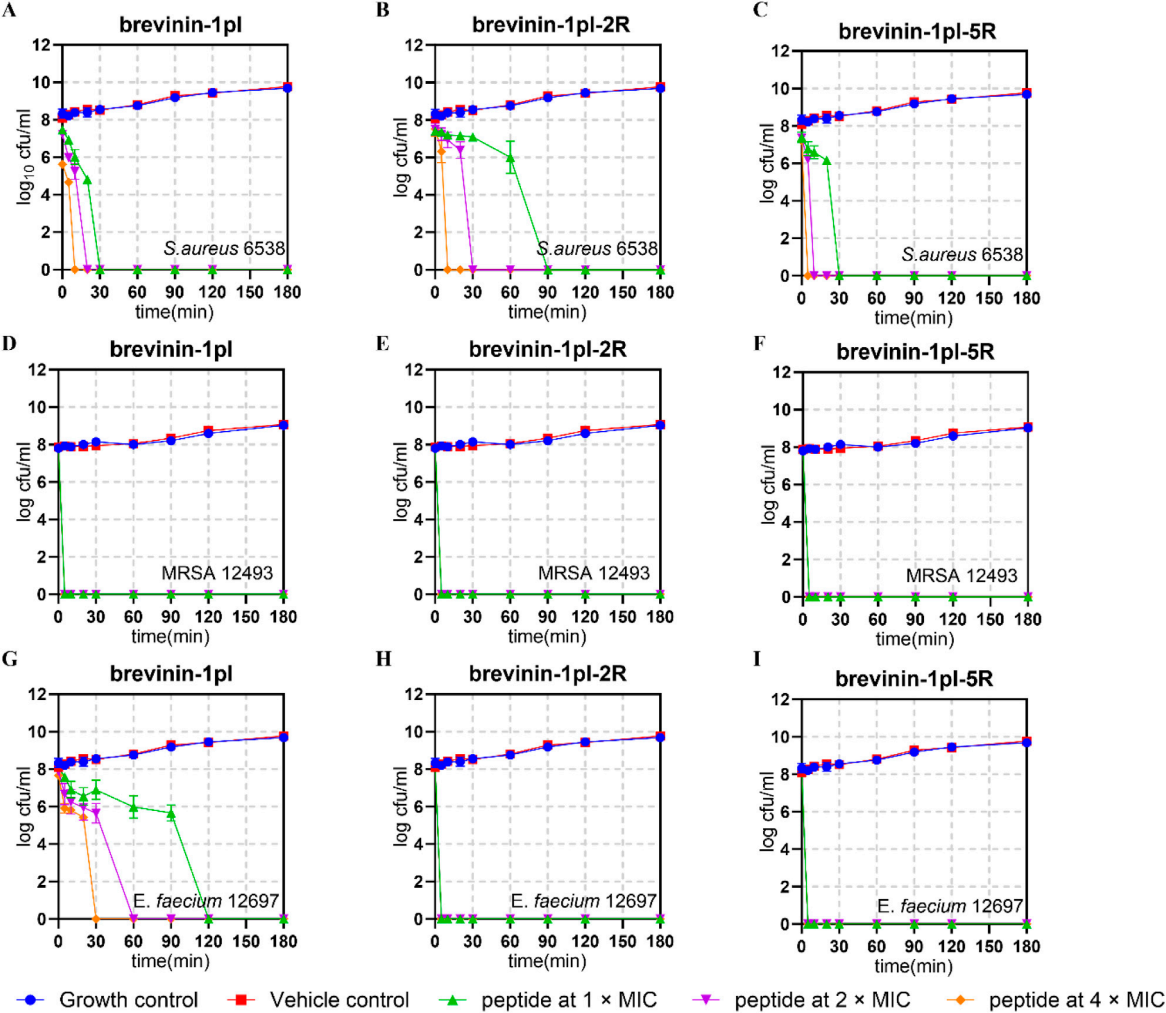
Time-killing kinetic curves of brevinin-1pl, brevinin-1pl-2R, and brevinin-1pl-5R against *S. aureus* 6538 **(A–C)**, MRSA 12493 **(D–F)** and **(E)** faecium 12697 **(G–I)**. In **(A–I)**, the MIC was 2 μM, while in Figures G and H, the MIC was 4 μM. The error bars represent the means ± SEMs for each independent experiment.

To study the bactericidal rate, bactericidal kinetics tests were conducted on Gram-positive bacteria, *S. aureus* 6538, *E. faecium* 12697, and MRSA 12493. The results are shown in Figure 5. All three peptides were able to completely inactivate *S*. *aureus* at the lowest inhibitory concentration within 180 min (Figures 5A–C). For MRSA 12493, all three peptides can immediately and completely kill the bacteria (Figures 5D,E). For *E*. *faecium*, brevinin-1pl-2R, and brevinin-1pl-5R showed almost immediate complete killing (Figures 5F–H).

### 3.6 Ability of brevinin-1pl and its analogues to influence membrane permeability of gram-positive and gram-negative bacteria

**FIGURE 6:**
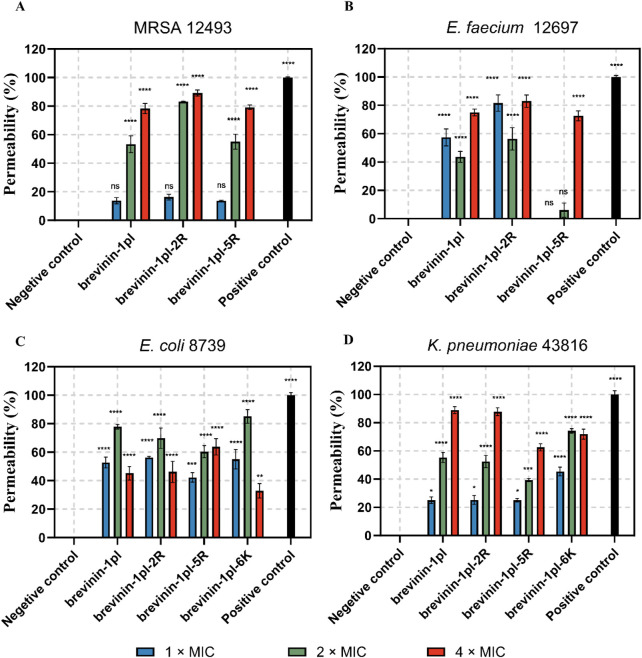
Effects of brevinin-1pl and analogues of brevinin-1pl the on the membrane of MRSA **(A)**, E. faecium **(B)**, *E. coli* **(C)**, and *K. pneumoniae* **(D)** strains. Changes in cytoplasmic membrane permeability in the presence or absence of peptides. Melittin is served as the positive control (Melittin = 100% lysis), and PBS was served as the negative control (PBS = 0% lysis). The error bars represent the means ± SEMs for each independent experiment. Asterisks represent **** P-value <0.0001, *** P-value <0.001, ** P-value <0.01, * P-value <0.5 compared to Negative control. The P-value represents the statistical significance of the observed differences.

To test the ability of the antimicrobial peptides to affect the permeability of membranes, Sytox-green assays were performed, and the results are shown in Figure 6. The results showed that the antimicrobial peptides could affect membrane stability for all the bacteria tested. The effect of peptides on the membrane of MRSA 12493 and K. *pneumoniae 43816* represent concentration-independent.

### 3.7 Anti-proliferative activities of brevinin-1pl and its analogues

**FIGURE 7:**
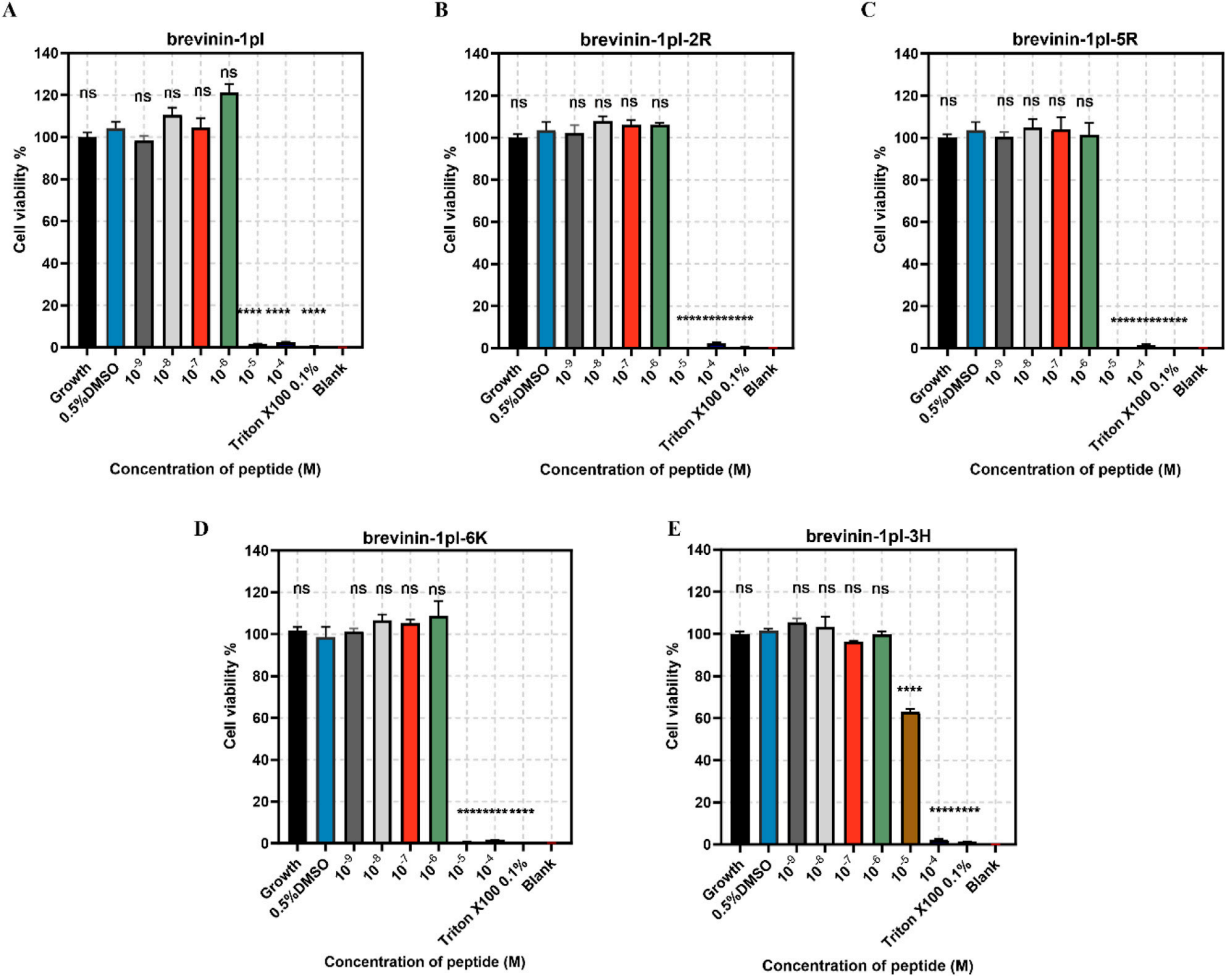
Anti-proliferation activity of peptide brevinin-1pl **(A)**, brevinin-1pl-2R **(B)**, brevinin-1pl-5R **(C)**, brevinin-1pl-6K **(D)** and brevinin-1pl-3H **(E)** on H838 cells. Cell lines treated with 0.1% Triton X-100, 0.5% DMSO and medium were used as the positive control, negative control and growth control. The blank group was treated with medium without cells. The error bars represent the means ± SEMs for each independent experiment. Asterisks represent **** P-value <0.0001 compared to 0.5% DMSO group. The P-value represents the statistical significance of the observed differences. Data were obtained from 9 replicates in three independent experiments.

**FIGURE 8:**
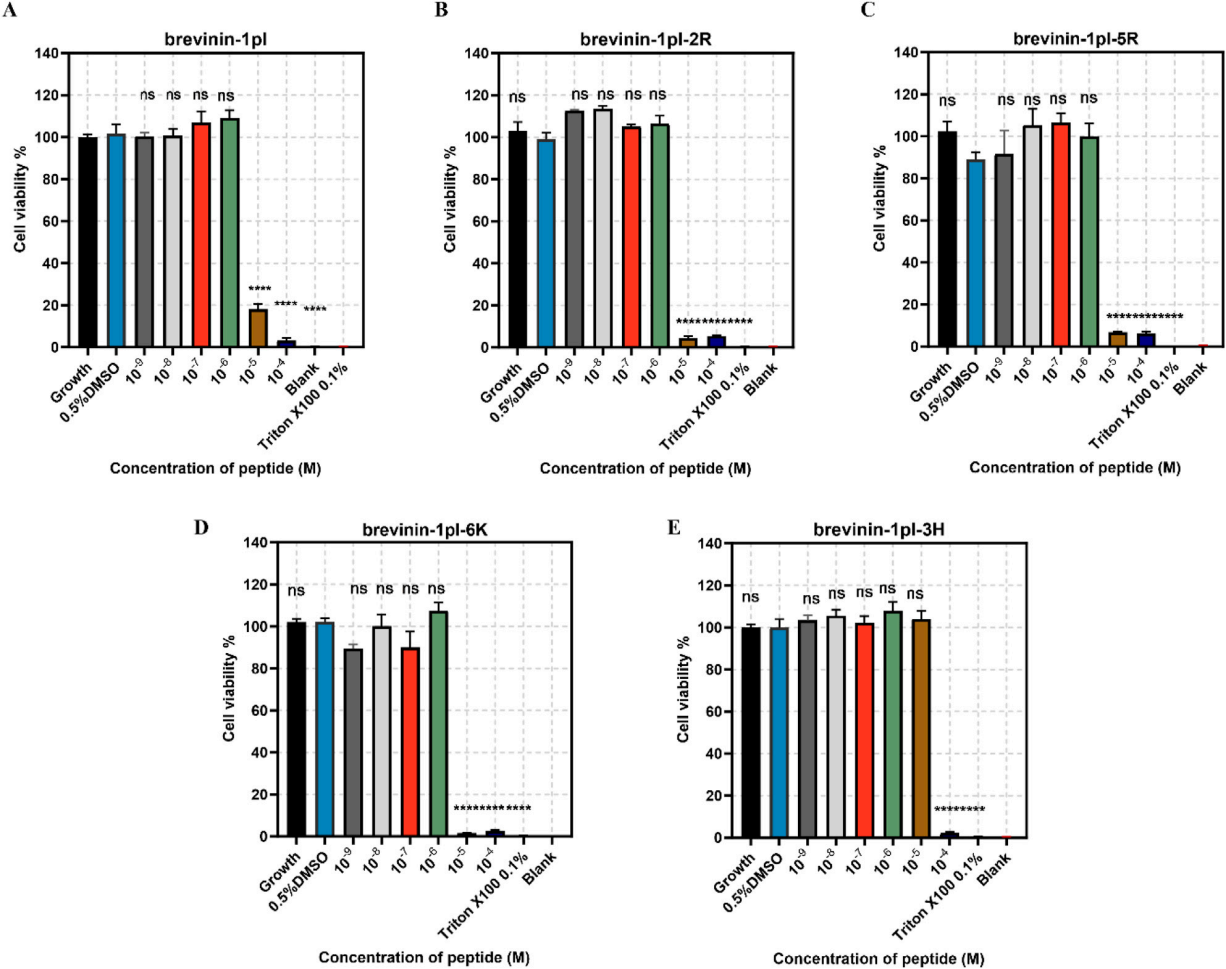
Anti-proliferation activity of peptide brevinin-1pl **(A)**, brevinin-1pl-2R **(B)**, brevinin-1pl-5R **(C)**, brevinin-1pl-6K **(D)** and brevinin-1pl-3H **(E)** on MCF-7 cells. Cell lines treated with 0.1% Triton X-100, 0.5% DMSO and medium, were used as the positive control, negative control and growth control. The blank group was treated with medium without cells. The error bars represent the means ± SEMs for each independent experiment. Asterisks represent **** P-value <0.0001 compared to 0.5% DMSO group. The P-value represents the statistical significance of the observed differences. Data were obtained from nine replicates in three independent experiments.

**FIGURE 9:**
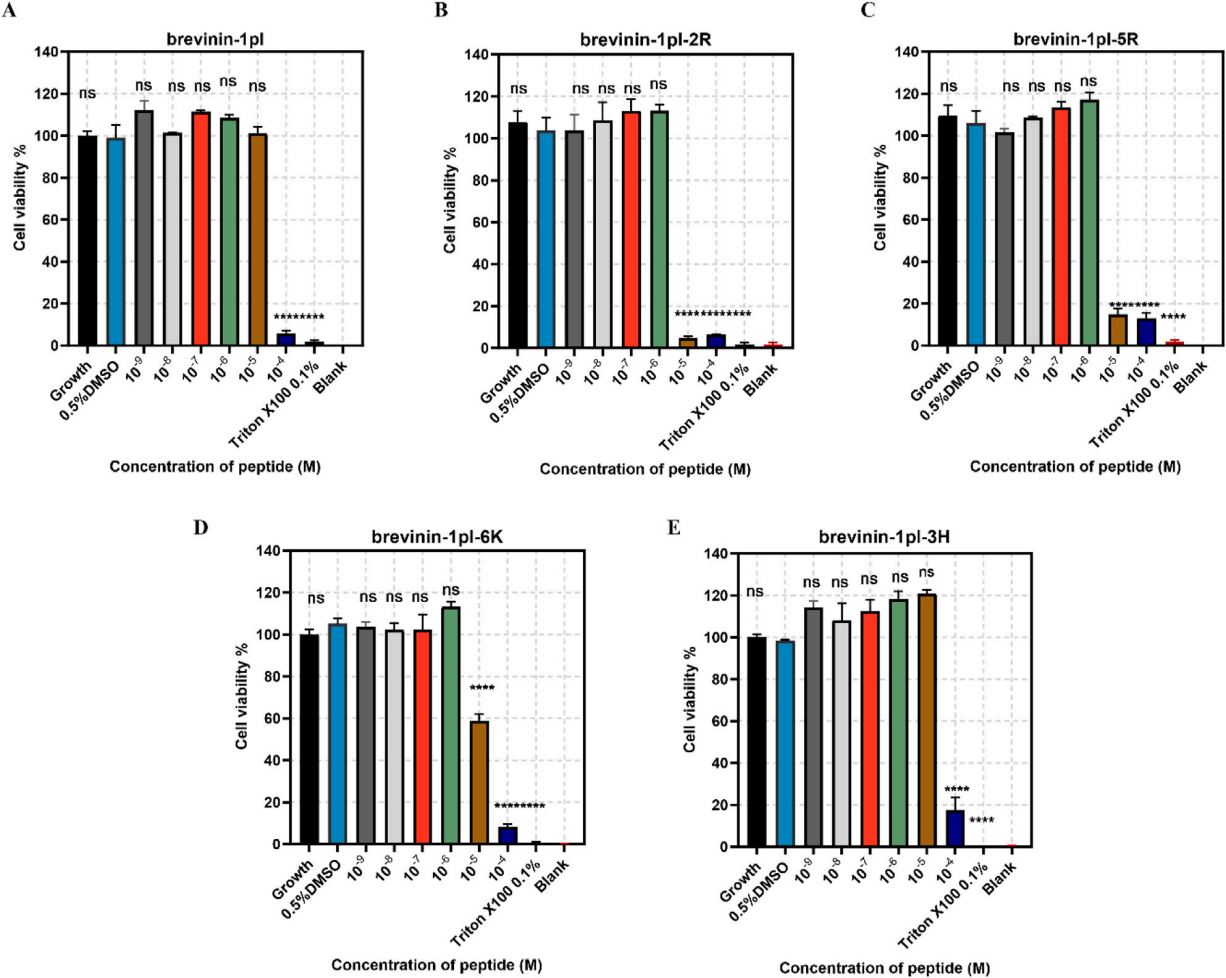
Anti-proliferation activity of peptide brevinin-1pl **(A)**, brevinin-1pl-2R **(B)**, brevinin-1pl-5R **(C)**, brevinin-1pl-6K **(D)** and brevinin-1pl-3H **(E)** on HaCaT cells. Cell lines treated with 0.1% Triton X-100, 0.5% DMSO and medium, were used as the positive control, negative control and growth control. The blank group was treated with medium without cells. The error bars represent the means ± SEMs for each independent experiment. Asterisks represent **** P-value <0.0001 compared to 0.5% DMSO group. The P-value represents the statistical significance of the observed differences. Data were obtained from nine replicates in three independent experiments.

Brevinin-1pl and its analogues were evaluated for their cytotoxic effects on both human cancerous cells and healthy cells. As shown in Figure 7, all of five peptides demonstrated significant inhibitory effects on the growth of H838 cell lines when the concentration exceeded 10^–5^ M. The antiproliferation activity of MCF-7 was shown in Figure 8, the brevinin-1pl-3H showed antiproliferation activity at the concentration of 10^–4^ M, other four peptide demonstrated antiproliferation activity at 10^–5^ M. For healthy cell HaCaT, brevinin-1pl and brevinin-1pl-3H demonstrate antiproliferation activity 10^–5^ M; brevinin-1pl-2R, brevinin-1pl-5R and brevinin-1pl-6Kdemonstrate antiproliferation activity at 10^–4^ M (showed in Figure 9).

## 4 Discussion

As a critical component of the host innate immune system, AMPs played a pivotal role in combating a wide range of microorganisms, driving extensive research into their potential applications as alternatives to traditional antibiotics (Duarte-Mata and Salinas-Carmona, 2023; Elbediwi and Rolff, 2024; Wang, 2014). Antimicrobial peptides derived from frog skin exhibit promising therapeutic potential (Kumari et al., 2020; Zohrab et al., 2019).

Previous studies had shown that brevinin-1pl possessed a distinctive structural architecture, characterized by a disulfide-bridged C-terminal “*Rana* box,” net positive charges, hydrophobic regions, and well-defined secondary structures, all of which synergistically caused its broad-spectrum antimicrobial properties (Wang et al., 2024). CD spectroscopy indicated that charge modifications do not disrupt its overall secondary structure, as it retained an alpha-helical conformation in TFE environments. This structure was essential for its ability to interact with and disrupt bacterial membranes (Huang et al., 2010). The α-helical secondary structure critically determines the activity of AMPs through amphipathic design and membrane-disruptive mechanisms. The helical conformation enables spatial segregation of cationic residues (lysine or arginine) on one face and hydrophobic residues (e.g., leucine, alanine) on the opposing face, creating an amphipathic topology essential for bacterial membrane interactions. This structural configuration facilitates electrostatic targeting, where cationic domains specifically bind to anionic bacterial membranes such as lipopolysaccharides on Gram-negative bacterial surfaces or teichoic acids on Gram-positive bacterial surfaces. Concurrently, the α-helical structure promotes membrane insertion, allowing hydrophobic regions to embed into lipid bilayers and induce curvature stress or pore formation via mechanisms like the “carpet” or “toroidal-pore” models (Bertelsen et al., 2023; Won et al., 2023). targeted electrostatic recognition and physical membrane disruption underpin the broad-spectrum antimicrobial efficacy of α-helical AMPs across diverse bacterial pathogens.

Antimicrobial results indicated that brevinin-1pl-2R replacing lysine with arginine in the “*Rana* box” did not significantly improve activity. Moreover, brevinin-1pl-5R replacing all lysine with arginine only slightly enhanced activity against Gram-positive bacteria but significantly reduced activity against Gram-negative bacteria, suggested that arginine’s penetration ability was more effective against Gram-positive organisms. Interestingly, peptide brevinin-1pl-6K with an added charge exhibited reduced antimicrobial activity against Gram-positive bacteria, indicating that increasing charge does not always enhance activity (Gagat et al., 2024). The associated decrease in hydrophobicity likely contributed to this decline in efficacy. Furthermore, brevinin-1pl-3H exhibited significantly diminished antimicrobial activity against gram-negative bacteria, as histidine did not generate additional charges at neutral pH, resulting in insufficient electrostatic interactions required for membrane targeting (Malik et al., 2016).

The structural dichotomy between Gram-positive and Gram-negative bacteria fundamentally dictates their antimicrobial susceptibility through distinct surface compositions. Gram-positive species feature thick peptidoglycan layers embedded with teichoic acids, whereas Gram-negative bacteria possess an outer membrane containing lipopolysaccharides (LPS) and porin channels, establishing differential permeability barriers. These architectural variations alter antimicrobial peptide penetration efficiency and target accessibility—the same peptides may exhibit distinct interaction patterns across bacterial surfaces. Beyond surface interactions, antimicrobial peptides may engage alternative binding sites, such as bacterial DNA recognition and binding, further diversifying their antibacterial mechanisms (Wang et al., 2024). The synergistic interplay of the aforementioned factors collectively governs the differential antibacterial efficacy of antimicrobial peptides across distinct bacterial species by modulating target accessibility and molecular interaction patterns.

From the hemolytic activity screening, brevinin-1pl-2R and brevinin-1pl-5R exhibit increased activity compared to the parent peptide, brevinin-1pl, which attributed to the number of arginine replacements and increased with the increase of replacement. This trend correlated with the number of arginine substitutions, suggesting that arginine enrichment enhances non-specific interactions with eukaryotic membranes, it also showed that the brevinin-1 peptide hemolysis mechanism is different from soshi-1 peptide (Yang et al., 2018). Meanwhile, this result is also similar to the findings from some studies on short peptides (Luong et al., 2018). The number of charges exerts a complex influence on both antimicrobial activity and hemolytic activity (Islam et al., 2023). The hemolytic activity of brevinin-1pl-6K and brevinin-1pl-3H showed that the introduction of a new arginine at the fifth site resulted in a reduction in hemolytic activity, and the substitution with histidine also led to a decrease in hemolytic activity. It suggested that balancing peptides’ charge and hydrophobicity can potentially enhance antimicrobial activity without increasing hemolytic effects (Roque-Borda et al., 2024). At the same time, the results of brevinin-1pl-3H indicate that deleting charges is also an effective approach to reducing hemolytic activity. Although its hydrophobicity is greatly improved, its hemolytic activity is reduced. If replaced with a simpler side chain alanine, it may be a practical option to reduce the hemolytic activity of peptides.

To investigate the advantages of arginine substitution, time-killing experiments were conducted against Gram-positive bacteria. The improved sterilization efficiency in clinical practice significantly shortens therapeutic duration and reduces the risk of iatrogenic infections (Rutala et al., 2023). Moreover, it optimizes clinical workflows to enhance patient comfort and treatment compliance. Furthermore, this investigation establishes a foundation for subsequent mechanistic studies by helping determine optimal experimental timeframes, thereby contributing to the systematic exploration of sterilization mechanisms and their clinical applications. The results revealed that for *S. aureus* 6538, complete substitution with arginine indeed enhanced the bactericidal rate, whereas the replacement of only two lysine with arginine reduced the bactericidal rate. This suggests that the lysine residues within the “*Rana* box” may contribute to additional bactericidal mechanisms. In contrast, for *E. faecium*, arginine substitution significantly increased the bactericidal rate, indicating that the introduction of arginine can enhance the peptide’s bactericidal efficacy against specific Gram-positive bacteria.

Next, SYTOX green assays were performed to investigate the effects of AMPs on cytoplasmic membrane permeability. Subsequent research efforts will prioritize optimizing antibacterial efficacy through mechanisms that circumvent excessive membrane disruption, as emerging evidence suggests a strong correlation between membranolytic activity and undesired hemolytic effects. Structural studies indicated that amphipathic motifs enabling pathogen membrane penetration may also interact non-specifically with erythrocyte membranes through analogous lipid-binding domains. Consequently, experimental strategies will focus on structural refinements that enhance target-selective binding to microbial components (e.g., teichoic acids or lipopolysaccharide) while preserving mammalian cell compatibility, guided by recent advances in structure-activity relationship modeling of antimicrobial peptides. The membrane disruption assays demonstrated that the parent peptide and its modified analogues exhibited strong membrane-disrupting capabilities, consistent with other reported peptides from the brevinin family (Abraham et al., 2015). However, the reduced activity of brevinin-1pl-6K against *E. faecium* and *E. coli* at higher concentrations may suggest the presence of non-membrane-related targets, though this hypothesis requires further validation through studies such as transcriptomic profiling or intracellular localization assays.

MTT assays were conducted to test the peptide cytotoxicity. In general, both high hydrophobicity and charge density contribute to the selectivity of AMPs toward bacteria and cells (Jiang et al., 2008). Experimental results demonstrated that brevinin-1pl and its analogues exhibited significant inhibitory effects on the H838 and MCF-7 cancer cell lines. Among them, brevinin-1pl-2R and brevinin-1pl-5R results showed that the introduction of arginine showed stronger inhibitory effects for HaCaT. The addition of a lysine residue to brevinin-1pl-6K resulted in enhanced inhibitory effects on normal HaCaT cells, indicating that an increase in charge may compromise the selectivity of AMPs toward normal cells. Interestingly, despite its high hydrophobicity, brevinin-1pl-3H did not exhibit increased inhibitory effects on HaCaT cells, but decreased inhibitory effects on H838 cells and MCF-7 cells, compared with the brevinin-1pl-6K, brevinin-1pl-3H require a certain number of charges to maintain their anti-proliferative activity. However, brevinin-1pl-3H with low hemolytic activity, low cytotoxicity and broad-spectrum antimicrobial properties, it holds potential for applications in post-surgical recovery and skin healing. There are many research directions on the inhibitory effect of antimicrobial peptides on cancer cells, such as membrane disruption intracellular targets and immunomodulation. Our antimicrobial peptide, due to its strong amphiphilicity, aqueous nature, and high charge, is speculated to have the main mechanism of action of electrostatic adsorption on the surface of cancer cells, and then use its amphiphilicity and hydrophobicity to bind to the cancer cell membrane and cause damage. Of course, there may be corresponding antimicrobial peptide binding sites on cancer cells that induce apoptosis, which is one of the directions for future research.

This study provides critical insights into the structure-activity relationship of brevinin-1pl and its analogues but highlights several limitations that necessitate further investigation. Firstly, the reliance on *in vitro* models fails to account for physiological complexities such as pharmacokinetic properties, systemic toxicity, or host-pathogen interactions in living organisms. Subsequent evaluations in animal models or clinical settings are essential to assess bioavailability, metabolic stability, and safety profiles. Secondly, the mechanistic basis of their antimicrobial action—particularly their specificity for bacterial membranes versus host cell membranes—remains incompletely resolved. Advanced methodologies, including high-resolution structural analyses (e.g., cryo-electron microscopy), membrane interaction assays, and omics-based approaches, are required to elucidate their mode of action and potential off-target effects. Thirdly, the variable efficacy observed across bacterial strains underscores the need to expand testing to clinically relevant, multidrug-resistant pathogens (e,g, MRSA) and biofilm-associated infections, which represent pressing therapeutic challenges. Finally, addressing formulation stability, scalability of synthesis, and long-term toxicity will be pivotal for clinical translation. Collectively, these gaps emphasize the necessity for interdisciplinary efforts integrating microbiology, pharmacology, and structural biology to advance brevinin-1pl analogues toward viable therapeutic applications.

## 5 Conclusion

This study systematically continued to investigate brevinin-1pl, an antimicrobial peptide derived from the skin secretions of *Rana pipiens*, designed several analogues using a set of bioinformatics tools to evaluate the effects of lysine, arginine, and histidine substitutions on its structural and functional properties. The results revealed that arginine substitution significantly enhanced hemolytic activity but did not markedly improve antimicrobial efficacy or membrane penetration capacity. In contrast, replacing polar amino acids with lysine reduced hemolytic activity while maintaining moderate bactericidal activity. The histidine modification results in a decrease in both antimicrobial and hemolytic activities, it also demonstrates potential for antifungal activity. These findings provide critical insights into the structure-activity relationship of brevinin-1 peptides, establishing a foundational framework for the rational design of next-generation antimicrobial agents. Notably, the analogue brevinin-1pl-6K emerges as a highly promising antibacterial candidate, demonstrating potent *in vitro* activity against a broad spectrum of multidrug-resistant Gram-negative pathogens. Its reduced hemolytic activity, coupled with enhanced selectivity for microbial membranes, underscores its potential for systemic or localized therapeutic applications. Concurrent optimization efforts yielded brevinin-pl-3H, which exhibits dual functionality: robust antimicrobial efficacy against dermatologically relevant pathogens (e.g., *Staphylococcus aureus*) and favorable biocompatibility with human epidermal keratinocytes (HaCaT cells). This dual-action profile, combined with pH stability under physiological conditions, positions brevinin-pl-3H as a compelling candidate for addressing polymicrobial skin infections, acne vulgaris, or chronic wound management. To advance these analogues toward clinical translation, future studies should prioritize *in vivo* validation of efficacy and safety in infection models, mechanistic elucidation of their immunomodulatory properties, and formulation optimization to enhance drug delivery and bioavailability. Moreover, exploring synergistic interactions with conventional antibiotics and resistance mitigation strategies is imperative to counteract evolving antimicrobial resistance (AMR) challenges. Collectively, this study bridges peptide engineering with therapeutic innovation, proposing two distinct research directions—broad-spectrum antibacterial therapy and targeted dermatological interventions to address global infectious disease burdens.

## Data Availability

The data presented in the study are deposited in the NCBI protein repository, accession number 2752633888 (https://www.ncbi.nlm.nih.gov/protein/2752633888).
